# Effects of a computerised guideline support tool on child healthcare professionals’ response to suspicions of child abuse and neglect: a community-based intervention trial

**DOI:** 10.1186/s12911-019-0884-y

**Published:** 2019-08-15

**Authors:** Annemieke A. J. Konijnendijk, Magda M. Boere-Boonekamp, Maria E. Haasnoot, Ariana Need

**Affiliations:** 1 Department of Health Technology and Services Research, Technical Medical Centre, University of Twente, Institute for Innovation and Governance Studies, P.O. Box 217, 7500 BK Enschede, the Netherlands; 2 Municipal Health Service GGD Twente, Department of Preventive Child Healthcare, P.O. Box 1400, 7500 BK Enschede, the Netherlands; 3 Department of Public Administration, Institute for Innovation and Governance Studies, University of Twente, P.O. Box 217, 7500 BK Enschede, the Netherlands

**Keywords:** Child abuse and neglect, Guideline adherence, Computerised support, Secondary prevention

## Abstract

**Background:**

Healthcare professionals’ adherence to guidelines on child protection is not self-evident. This study assessed the effects of a computerised support tool on child healthcare professionals’ adherence to the seven recommended guideline activities, and on time spent seeking information presented in this guideline.

**Methods:**

A community-based intervention trial design was applied, comparing access to a paper-based guideline (control) with access to a paper-based guideline supplemented with a computerised guideline support tool (intervention). A total of 168 child healthcare doctors and nurses working in one large Dutch organisation were allocated to an intervention or control group. Outcomes were professionals’ performance of seven recommended guideline activities and the amount of time spent seeking information presented in the guideline. Professionals’ adherence was measured using two methods: health record analysis and a self-report questionnaire. The questionnaire was also used to collect data on the amount of time spent seeking guideline information.

**Results:**

In total, 152 health records (102 in the intervention group and 50 in the control group) were available for analysis. The tool was registered in 14% of the records in the intervention group. Performance of activities, corrected for intentional non-adherence, was except for one activity, high (range 80–100%); no differences were found between the control and intervention groups. Forty-nine questionnaires (24 in the intervention group and 25 in the control group) were analysed. Sixty-three percent of the questionnaire respondents (15/24) claimed to have used the tool. No differences in guideline adherence were found between the two groups. Respondents in the intervention and control groups spent, on average, 115 and 153 min respectively seeking relevant information presented in the guideline.

**Conclusions:**

The results regarding use of the tool were inconclusive as the outcomes differed per method. In contrast to expectations, performance of guideline activities was high in both groups. The support tool may decrease the amount of time spent on seeking guideline information. However, given the high adherence scores and small number of questionnaire respondents, the outcomes failed to reach statistical significance. Future research should focus on studying the effects of the tool after a longer period of availability.

**Electronic supplementary material:**

The online version of this article (10.1186/s12911-019-0884-y) contains supplementary material, which is available to authorized users.

## Background

Child Abuse and Neglect (CAN) has been recognised worldwide as a serious public health issue [1] that needs to be prevented. As abusive parents, maltreated children, and bystanders frequently do not seek help [2, 3], national policies have increasingly emphasised the responsibility of healthcare professionals, sometimes statutorily [4–6]. However, multiple studies have shown that healthcare professionals under-identify cases of CAN or do not always respond adequately to concerns (e.g. [7–11]). As poor recognition and response may result in continued CAN, potentially leading to severe consequences [1], health professionals should be supported around these issues.

One approach to support professionals and improve handling of CAN concerns is to provide them with evidence-based guidelines. In the last decade, guidelines on responding to suspected CAN have increasingly become available [12–14], including in the Netherlands. Since July 2013, Dutch professionals involved with children and families have been legally obliged to follow guidelines if they suspect CAN [15]. In 2010, the Dutch Centre for Child Health issued a clinical guideline on early detection and response to suspected CAN (further referred to as the CAN guideline) [16, 17]. This guideline has been developed specifically for preventive child healthcare (CHC) doctors and nurses. These professionals are tasked with the identification and registration of (potential) risks to the health and wellbeing of children. The developers of the guideline listed seven key activities which they deem critical, based on an analysis of scientific literature and consensus among professionals and experts [17]. The guideline, which was made available in a paper-based format, is 170 pages long and is also available online. A 40-page summary, a two-page document listing the key activities and a flowchart were also made available to CHC professionals. The key activities described in the CAN guideline are shown below, presented in the order in which the guideline recommends CHC professionals perform them:

- Risk assessment based on protective and risk factors;
- Discussing suspicions with caregiver(s) and/or child;
- Consulting an in-house expert on child abuse and neglect;
- Consulting the regional child protection service: the Advice and Reporting Centre;
- Requesting information from professionals outside the child healthcare organisation who are also involved with the family;
- Acting: providing support, referring the family to other organisations for support or reporting suspicions to the Advice and Reporting Centre;
- Monitoring the support that is provided to the family and taking action again if the support is inadequate.

Despite the potential benefits, including more consistent work procedures based on the best available evidence and improved health outcomes [18], adherence to guidelines is generally poor (e.g. [19–21]). Two studies have evaluated CHC professionals’ performance of CAN guideline activities. Fleuren et al. [17] documented a range of performance of five key activities varying between 67 and 82%. Konijnendijk et al. [22] reported that the percentage of professionals that performed a key activity in all suspected cases varied between 19.5 and 42.7% for the seven activities.

One approach to promote routine use of guidelines is to introduce a computerised guideline support tool through which guideline information is disseminated in a more user-friendly manner [21]. Paper-based guidelines have been criticised for quickly becoming outdated, and being a suboptimal presentation format [23]. Communicating guidelines through a computerised system with an interface similar to an electronic health record makes the application of guidelines more personal and acceptable at the moment of care [24]. Health Information Technology (IT) systems, such as electronic health records, and computerised guideline support tools, are increasingly being introduced in healthcare organisations to enhance care efficiency, quality, and safety [25–27]. The need for health IT to facilitate use of guidelines has been discussed in literature [21, 23, 28]. Previous research has shown that a support tool can improve healthcare professionals’ adherence to guidelines and improve professional practice [29, 30].

We developed a computerised guideline support tool that was integrated in the electronic health record used in Dutch preventive CHC. The tool presents guideline information in a concise manner that is quickly and easily accessible. Guideline information is offered at the time and place of decision-making, which has been described as best practice with regard to design principles for usable decision support [31]. The tool also presents record data regarding risk factors. Furthermore, the tool instructs professionals to plan appointments or to perform tasks within the timeframe that the guideline recommends. Also, it reminds professionals to perform activities and register these activities in the health record by use of electronic alerts, and provides real-time notifications when activities are not performed in time. Electronic reminders may achieve small to modest improvements in clinical behaviour [32]. Overall, the tool aims to promote guideline adherence and uniform registration, and to minimise the time and effort needed to access guideline information. As such, the tool may increase both the quality of healthcare and professional productivity. The development of the tool, which is described elsewhere [33], followed an iterative process involving CHC professionals in all developmental stages. Addressing professional attitudes towards a computerised support tool has been shown to be important for its successful implementation [24, 34].

This study addressed the following question: What are the effects of having access to the paper-based CAN guideline complemented with a computerised guideline support tool, compared to having access solely to the paper-based version, on CHC professionals’ adherence to the guideline and on the amount of time spent seeking relevant information provided by the guideline? In this study, the adherence scores were corrected for intentional non-adherence. Arts et al. [21] concluded in their recent systematic review that deviations from guidelines are often supported by valid reasons and that these intentional deviations can also result in good quality of care.

## Methods

### Design

A mixed-methods community-based intervention trial [35] design was applied, comparing access to a paper-based guideline complemented with a computerised guideline support tool (intervention) with access to a paper-based guideline only (control). The study was carried out in one large Dutch CHC organisation (GGD Twente) in the eastern part of the Netherlands.

### Setting

In the Netherlands, CHC professionals provide routine preventive services to virtually all children (0–17 years) in well-baby clinics and schools. These services concentrate on the optimal growth and development of a child, to prevent the child from developing severe health problems [36]. Except for short-term parenting support, CHC services do not provide treatment [9]. In the Netherlands, CHC professionals are well placed to detect, monitor, and respond to suspected CAN for two reasons. First, they have frequent contact with families and their children for consultation, usually 15 times between the ages of zero and four and five times after the age of four [37]. Second, both disciplines have been trained extensively to develop skills in recognising and managing health, psychosocial, and parenting problems. Doctors and nurses work together daily and intensively in a CHC team. They use an electronic health record to keep track of the child’s development.

### Study population and group allocation

The subjects of this study are CHC professionals working at GGD Twente. In January 2014, GGD Twente employed 58 CHC doctors and 110 CHC nurses, divided over 21 teams. Professionals in the same CHC team provide services to the same children in a specific geographically demarcated region, such as a village or a city district. All professionals were female. During the study period (February 2014 – October 2014), GGD Twente provided services to approximately 125,000 children between 0 and 17 years old [38].

All 168 CHC professionals were allocated to one of two groups. When assigning professionals to a group, we addressed three issues:

- Professionals who provide care to the same children, and thus document client information in the same health records, should be in the same group;
- Contamination between professionals in the intervention and the control group should be minimised [39]. The risk of contamination is expected to be lower when allocation to the two groups is performed on a team level. Twenty-three professionals were part of two or more CHC teams. These professionals should preferably be in the same group to prevent contamination;
- Both groups had to represent urban and rural areas in equal proportions.

After making the best possible division of teams into two groups, taking into consideration the issues mentioned above, the two groups were randomly assigned to the intervention (11 CHC teams) or the control groups (ten CHC teams) by flipping a coin. Unfortunately, we could not prevent four professionals being assigned to both conditions.

#### Intervention group

The intervention group comprised 90 professionals, including the four professionals in both groups: 32 doctors and 58 nurses. These professionals provide services to approximately 60,000 children [38]. Prior to the start of the study, all professionals in the intervention group were informed about the aim and procedure of the study in a meeting, and instructed in the use of the tool via a manual and a web-link with an online instruction video. They were asked to use the tool from February 1st, 2014 onwards. A helpdesk, comprising the first author and one of the application administrators, was available during the study period to assist professionals who had questions or encountered problems using the tool.

A professional with suspicions of CAN could access the tool in the electronic health record and link it to a child’s record. The tool has the following functionalities:

- presenting guideline information in a concise manner that is easily and quickly accessible;
- presenting guideline information at the time and place of decision-making;
- providing professionals with (up-to-date web links to) a clear overview of information, aids, and instructions on how to register information about a child’s situation correctly (i.e. accurate, complete, and uniform);
- displaying relevant information tailored to the organisational and regional context, such as contact details of the in-house CAN expert and professionals from other organisations involved with the family;
- displaying relevant data registered elsewhere in the child’s health record, including present risk factors;
- providing instructions to plan appointments or perform tasks guideline activities within the timeframe that the guideline recommends;
- reminding professionals to perform activities and providing real-time notifications when activities are not performed in time, through use of electronic alerts. These prompts are initiated by registrations that the professionals made earlier.

**Fig. 1:**
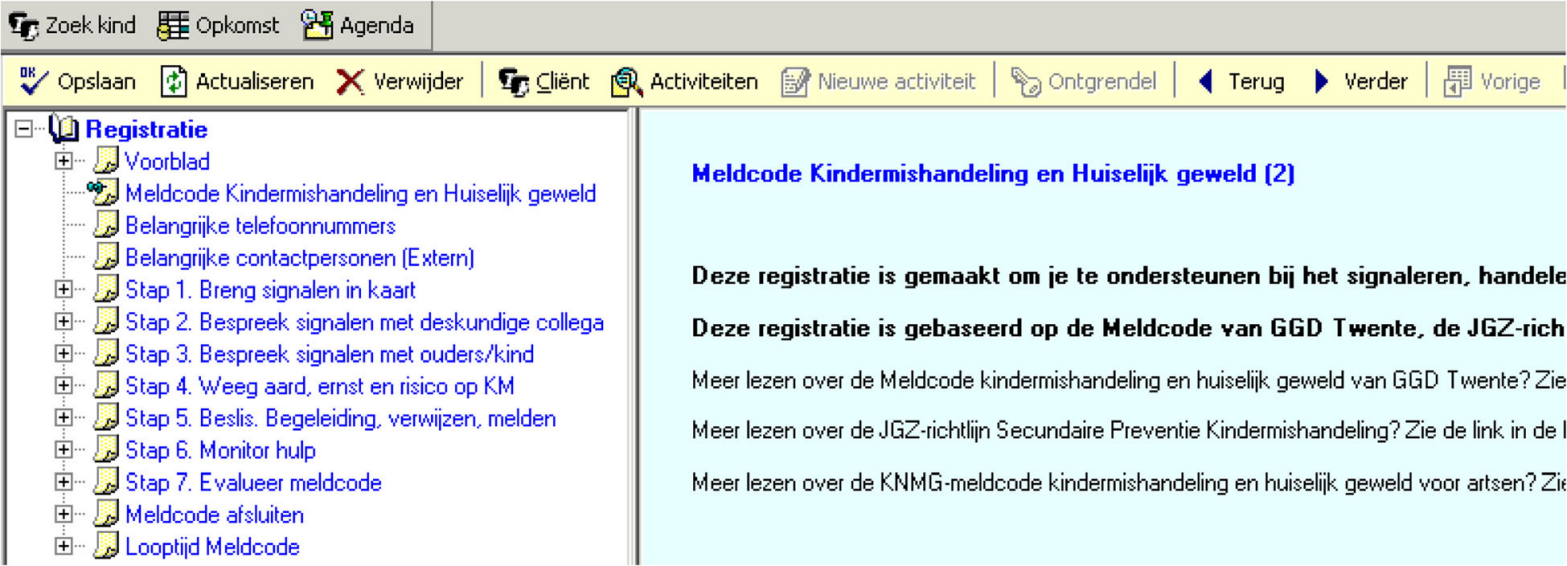
Screenshot of the tool: tab containing overview page

Figure 1 displays a screenshot of the overview page of the tool, which shows the clinical pathway on the left side: the activities that CHC professionals should perform, sequenced in the most common and logical order.

#### Control group

The control group consisted of 78 professionals: 26 doctors and 52 nurses. These professionals provide services to approximately 65,000 children [38]. They did not receive any instructions regarding the tool, but were informed about the study.

### Measurements

Two outcomes were measured: professionals’ adherence to seven recommended guideline activities and time spent seeking information presented in the guideline. Two methods were used to measure professionals’ adherence: record analysis and a self-report questionnaire. The survey was also used to collect data on the time spent seeking guideline information.

The adherence scores were corrected for intentional non-adherence [21]. Valid reasons for non-adherence included: ‘no longer had suspicions’, ‘a team member performed this activity’, ‘caregiver(s) or the child did not approve’ (applies to the activity ‘requesting information from other professionals outside the CHC organisation who are also involved with the family’), ‘a professional outside the organisation informed the CHC professional’ (applies to the activity ‘monitoring actual execution of an activity promoted by the guideline), and ‘a professional outside the organisation performed the activity or is responsible for performing the activity as (s)he was the first professional addressing concerns of possible CAN’. The reason ‘a team member performed this activity’ did not apply to record analysis, as these records were analysed on a team level.

### Health records

**Fig. 2:**
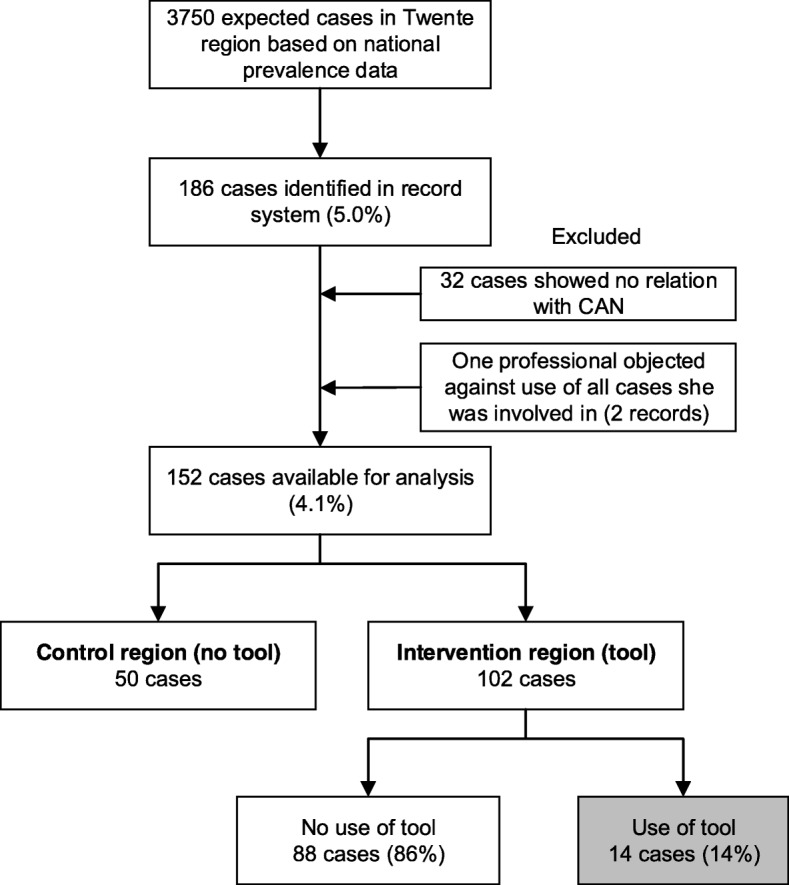
Flow diagram of the selection of health records

In total, the health records of 186 children met the following inclusion criteria: 1) The record has a registration that indicates suspected CAN; 2) The first registration indicating a suspected CAN case was made between February 1st and October 1st 2014. Two cases were excluded because one CHC professional objected to the use of her (anonymised) record data and 32 cases were excluded as content analysis revealed that the situation described in the record was unrelated to CAN. In total, 152 health records were available for analysis: 50 from the control group and 102 from the intervention group. Based on national data on the prevalence of child abuse and neglect [40], it was expected that 3750 children in the study region experience child abuse and/or neglect. Figure 2 displays a flow diagram of the record selection.

The cases described in the health records were followed for 8 months, starting from the date of the first registration that indicated suspicions of CAN. Personal data in the records were anonymised at both the child and the professional level by the application manager and the second author (RH). Afterwards, the first author (AK) read each record completely and coded information regarding adherence to the seven guideline activities, and gender, age of the child, and use of the tool.

#### Adherence to key guideline activities

For each case, yes/no scores were used to indicate whether each guideline activity was adhered to, corrected for intentional non-adherence [21].

#### Other variables

Gender and age of the child were documented for each record. Furthermore, use of the tool was analysed for each record. Use of the tool was assumed when the tool was registered in the record. Subsequently, information about the way suspicions arose was retrieved from the records: through observation, through a story from the child, a story from the caregiver(s), a story from a fellow CHC professional in the same organisation, the story of a professional outside the CHC organisation who is also involved with the child, or through the story of a non-professional (e.g. grandparent or neighbour). In 55 out of the 152 cases, CHC professionals were the first professional involved with the child who addressed concerns of possible CAN: 17 in the control group and 38 in the intervention group.

### Questionnaire

The questionnaire included questions concerning background variables, performance of key guideline activities, and amount of time spent seeking information from the CAN guideline (Additional file 1). For all questions, respondents were asked to keep the last suspected case in mind (between February 1st and October 1st 2014). Respondents in the intervention group who reported having used the tool at least once were asked to keep in mind the last suspected case for which they had used the tool. Because the last suspected case could have started, for example, in September 2014, it is possible that respondents had not performed all guideline activities before October 1st. Therefore, if a survey respondent mentioned that she did not perform a guideline activity because she had not yet got to it before October 1st, this answer was coded as a valid reason for non-adherence.

The questionnaire emphasised anonymity and confidentiality. A pilot-test, with two researchers and a CHC professional, was performed to assess the comprehensibility and practicality of the questionnaire. Subsequently, the questionnaire was digitalised using the online survey program LimeSurvey. All CHC professionals were invited, by email, to participate in the questionnaire in November 2014. In the second week after the initial mailing, a first reminder was sent. A second reminder was sent a week before closing the database in January 2015.

**Fig. 3:**
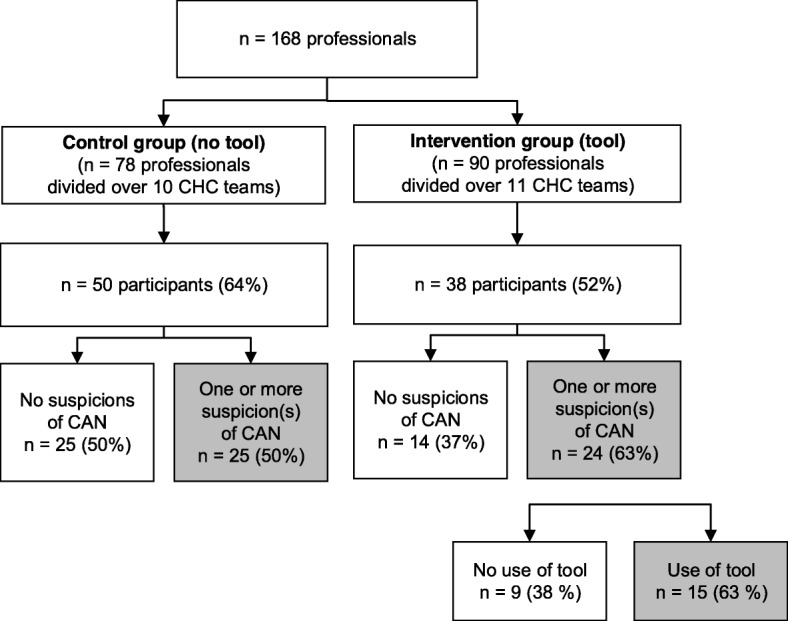
Flow diagram of questionnaire participants per group

Eighty-eight professionals filled in the questionnaire (52%). Subsequently, 39 respondents (44%) were excluded because they did not have suspicions of CAN between February 1st and October 1st 2014. In total, 49 questionnaires were available for analysis: 25 in the control group and 24 in the intervention group. Figure 3 displays the flow of professionals participating and eligible for the survey study.

#### Adherence to key guideline activities

The questionnaire was used to investigate whether, and how often, CHC professionals self-reportedly performed each key activity in the CAN guideline in their last suspected case. If respondents did not perform a key activity, they were asked to report why they had not performed the activity. If a reason was regarded as valid, non-adherence was recoded to ‘adherent’.

#### Time spent on seeking information provided by the CAN guideline

All respondents were asked to indicate the number of minutes they had spent on seeking relevant information provided by the paper-based guideline and/or the tool. In addition, respondents in the intervention group were asked to estimate how many minutes they had invested in learning to use the tool.

#### Other variables

Respondents were asked to report background variables including their profession, their number of working hours per week, and their years of experience as a CHC professional. As in the record analysis, data with regard to the way suspicions arose and use of the tool were collected.

### Data analysis

All analyses were performed using the intention-to-treat approach [41]. This approach allows for non-adherence to the tool by professionals in the intervention group. All records and survey participants were included in comparative analyses, regardless of whether they actually reported having used the tool.

#### Health records

Record data were entered manually into SPSS, version 24. The second author (RH) checked the analysis of 20 randomly selected records (11%), and did not identify inconsistencies or mistakes. Descriptive analyses were performed to describe both background information and outcome variables per group. Subsequently, independent sample t-tests were carried out to compare adherence scores between groups. *P*-values of <.05 (two-tailed) were considered significant.

#### Questionnaire

Questionnaire data were downloaded from LimeSurvey [42] to SPSS. For each condition, descriptive analyses were performed on background variables, adherence to guideline activities, and time spent seeking information provided by the guideline. A chi-square goodness of fit test was performed to test whether the observed proportions for discipline differ from the hypothesised proportion (35% doctors and 65% nurses). Chi-square tests and independent sample t-tests were carried out to identify differences regarding background and outcome variables between the two conditions (two-tailed). Statistical significance was defined as a *p*-value less than .05 (two-tailed). The data related to time spent seeking information were not normally distributed. Therefore, a non-parametric test was performed (Mann-Whitney test) to test differences between the control group and the intervention group.

## Results

### Background characteristics

The children (79 girls and 73 boys) whose records were included were on average 6.5 years old (SD = 5.2, range 0–17). The records in the intervention group (*n* = 102) on average concerned older children (M = 7.3 years old, SD = 5.1) than the records in the control group (*n* = 50; M = 5.1 years old, SD = 5.2) (*p* = .01).

The descriptive statistics for the background variables of questionnaire respondents with suspicions of CAN are presented in Table 1. Most of these respondents were nurses (67%), as expected based on the proportion of nurses and doctors in the organisation (χ^2^ (1) = 12, *p* = .73). Furthermore, most respondents provided services in well baby clinics (49%), 33% provided school healthcare, and 18% provided both services. The respondents had on average 19.5 years of work experience (SD = 8.3) and worked on average 21.7 h per week (SD = 5.0). The 49 participants with suspicions of CAN had on average 2.3 suspicions of CAN. No significant differences in background variables were found between the groups.

**Table 1:** Background variables of questionnaire respondents with suspicions of CAN in the previous 12 months (*n* = 49)

Characteristic	Participants with suspicions of CAN (*n* = 49)		Control group (10 CHC teams, *n* = 25)		Intervention group (11 CHC teams, *n* = 24)		
	n	%	n	%	n	%	
Profession							
Nurse	33	67	15	60	18	75	χ2 (1) = 1.25, *p* = .26
Doctor	16	33	10	40	6	25	
Service provision							
Well baby clinic	24	49	13	52	11	46	χ2 (2) = 1.40, *p* = .50
School healthcare	16	33	9	36	7	29	
Both services	9	18	3	1	6	25	
	n	M (SD)	n	M (SD)	n	M (SD)	
Years in professional practice	49	19.5 (8.1)	25	21.0 (8.0)	24	18.0 (8.3)	t (47) = 1.33, *p* = .19
Working hours per week	48	21.7 (5.0)	24^a^	22.0 (5.3)	24	21.5 (4.8)	t (46) = 0.34, *p* = .73
Number of suspicions	49	2.3 (1.3)	25	2.5 (1.2)	24	2.1 (1.3)	t (47) = 0.98, *p* = .33

^a^ *n* = 24 because of one missing value

### Use of the tool

In the intervention group, the tool was used in 14 out of the 102 records (14%). According to the questionnaires, 15 out of 24 respondents (63%) reported having used the tool in the intervention condition.

### Adherence to key guideline activities

Neither the record analysis nor the questionnaire analysis showed significant differences in adherence scores between the respondents in the control group and the intervention group. Table 2 shows adherence scores based on the record analysis and on the questionnaire data. Self-reported adherence rates were on average high. Except for the activity ‘risk assessment’ and the activity ‘in-house consultation of the CAN expert’, these outcomes are consistent across the two methods used. Risk assessment was virtually never registered in the records, while all respondents in the questionnaire reported that they had performed this activity in the suspected case they answered questions about. Adherence to consultation of an in-house expert on CAN was on average less frequently reported in the records (84%) than in the questionnaire (96%).

**Table 2:** Adherence to key CAN guideline activities (%, n) per method

Key guideline activities	Health records			Questionnaire		
	Control region (*n* = 50)	Intervention region (*n* = 102)		Control group (*n* = 25)	Intervention group (*n* = 24)	
1. Risk assessment based on protective and risk factors	6% (3)	3% (3)	t(150) =0.91, *p* = .37	100% (25)	100% (24)	–
2. Discussing suspicions with caregiver(s) and/or child	98% (49)	100% (100)	t(150) = −0.16, *p* = .99	100% (25)	100% (24)	–
3. Consulting an in-house expert on child abuse and neglect	80% (40)	85% (87)	t(150) = −0.79, *p* = .41	96% (24)	96% (23)	t(47) = 0.03, *p* = .98
4. Consulting the regional child protection service: the Advice and Reporting Centre	82% (41)	81% (83)	t(150) = 0.09, *p* = .93	84% (21)	96% (23)	t(47) = −1.37, *p* = .18
5. Requesting information from professionals outside the child healthcare organisation who are also involved with the family	96% (48)	93% (95)	t(150) = 0.70, *p* = .49	100% (25)	96% (23)	t(47) = 1.00, *p* = .33
6. Acting: providing support, referring the family to other organisations for support or reporting suspicions to the Advice and Reporting Centre	100% (50)	98% (99)^a^	t(150) = 1.42, *p* = .16	100% (25)	100% (24)	–
7. Monitoring the support that is provided to the family and taking action again if the support is inadequate	88% (44)	83% (84)^a^	t(149) = 0.77, *p* = .44	96% (21^b^)	100% (23^c^)	t(43) = −1.00, *p* = .33

^a^ *n* = 101 because of one missing value; ^b^ *n* = 21 because of 3 missing values; ^c^ *n* = 23 because of one missing value

In this study, valid reasons for non-adherence included ‘a professional outside the organisation performed the activity or is responsible for performing the activity as she was the first professional to raise concerns of possible CAN’. Therefore, we also analysed only the 55 records (17 in the control condition and 38 in the intervention condition) of cases in which a CHC professional was the first to raise concerns. In this analysis, the adherence scores were considerably, but not significantly, lower for two activities compared to the analysis of all 152 records: ‘Consultation of the in-house CAN expert’ (47% in the control condition and 76% in the intervention condition; *p* = .05) and ‘Consultation of the Advice and Reporting Centre’ (65% in the control group and 66% in the intervention group; *p* = .94).

### Time spent seeking information provided by the CAN guideline

Respondents in the intervention group spent significantly less time on the use of paper-based guidelines compared with the control group, respectively 75 min (SD = 54 min) and 135 min (SD = 121 min) (*p* = .01). The respondents in the intervention group spent an average of 40 min using the tool (SD = 47 min). When the time spent on the use of the paper-based guideline is combined with the time spent on the use of the tool to seek guideline information, the difference between the control group (M = 153 min, SD = 121 min) and the intervention group (M = 115 min, SD = 80 min) is still considerable, but not statistically significant (*p* = .34). Respondents in the intervention group had to invest time in learning how to use the tool. Respondents in the intervention group spent on average 53 min (SD = 42 min) on learning to use the tool.

## Discussion

This study assessed the effects of integrating a computerised guideline support tool into the electronic health record on CHC professionals’ adherence to a guideline on the early detection and response to suspected CAN, and on time spent seeking information presented in this guideline.

Assuming that 3% of the 125,000 children living in the study area experience CAN [40], the number of identified records of suspected cases (152) is much lower than expected. The identification of suspected CAN cases in the electronic health record depended on structured data. Structured data refers to registrations of fixed names for interventions and activities that indicate cases of CAN, such as ‘(suspected) CAN’, ‘worrying situation’, or the registration of the tool. The low number of identified cases may be due to professionals’ preferences around registering suspicions of CAN in the electronic health record using these fixed registrations [43], as CAN is a poorly defined condition, the ‘diagnosis’ is uncertain, and professionals are often ambivalent about using a label that explicitly refers to CAN [44]. It is possible that, as in the United Kingdom [43, 44], Dutch CHC professionals use a wide variety of phrases to indicate suspected CAN, including those that are indirect or euphemistic. As a result, using structured records to identify CAN is likely to result in an underestimation of suspected CAN cases.

The number of identified cases was especially low in the control group. Although the allocation of CHC teams to the two groups was performed carefully, it was remarkable that two-thirds of the CAN records were retrieved from the intervention group and that children in the intervention group were on average 2 years older. More professionals were assigned to the intervention group (90 compared to 78 in the control condition) due to the uneven number of teams that had to be allocated. However, it is not likely that the small difference in the number of professionals in each group explains why twice as many cases were identified in the intervention group. It is also improbable that CAN is more prevalent in the intervention region. Data on registered incidents of domestic violence (including CAN) in the study area in 2014 showed that 51% of these incidents took place in the intervention area [45]. Possibly (training in) using the tool in the intervention group helped to raise both awareness of CAN and attentiveness to possible indicators of it and stimulated professionals in the intervention group to register CAN-related concerns in the electronic health record.

### Use of the computerised guideline support tool

The two investigation methods used show inconclusive results regarding use of the tool in the intervention group: 63% (questionnaire) versus 14% (records). There are several possible explanations for these inconsistent percentages. On the one hand, the high usage rate using the survey method may be due to a self-selection bias [46] and social desirability bias [47]. Professionals who used the tool may have been more inclined to participate in the survey and survey respondents may have provided socially desirable answers. These biases probably resulted in an overestimation of the actual use percentage.

In conclusion, actual use of the tool probably lies between 14 and 63%, showing that many professionals did not adopt use of the new tool. Much literature has described the problem of lack of acceptance of technology in the healthcare field and has sought to predict and explain why some individuals adopt while others reject an innovation [48–51]. Perceived usefulness and perceived ease of use predict a substantial proportion of use or acceptance of health information technology (IT) [48, 51]. Compatibility between health IT and clinical work, individual characteristics, and organisational culture can also influence health IT acceptance [26, 51]. Furthermore, as the time between introduction and evaluation was short in the current study (8 months), innovators and early adopters may have been more likely to use the tool [49]. Use of the tool may be higher if a longer time frame had been chosen between introducing the tool and evaluating its use.

### Adherence to key guideline activities

The findings demonstrate that adherence to recommended guideline activities, corrected for intentional non-adherence, was high in both the intervention and control groups. These results were consistent between methods for five out of the seven guideline activities. The activity ‘Risk assessment based on protective and risk factors’ was virtually never identified in the records (intervention and control condition), while all 49 survey respondents mentioned that they performed this activity. These inconsistent results indicate that professionals either do perform risk assessment when they suspect CAN but do not register this activity explicitly in the child’s record or that they provided socially desirable answers in the survey [47]. The adherence score for the activity ‘Consulting an in-house expert on CAN’ was also higher in the survey than in the record analysis. The results do not provide evidence that the support tool improves professionals’ adherence to the CAN guideline.

This study shows that adherence to guideline activities, corrected for intentional non-adherence, was higher than expected based on previous research that did not correct for valid reasons not to perform guideline activities [17, 22]. Non-adherence to guidelines on CAN prevention can be supported by justifiable and thus valid reasons, in concordance with the findings of Arts et al. [21]. It is important to distinguish between reasons for non-adherence that are intentional and non-intentional. Insight into reasons for non-adherence that are not justifiable, such as poor knowledge, fear of consequences, or low confidence in follow-up care [10, 22, 52], provides opportunities for improvement in the quality of care for vulnerable children. Moreover, it makes clearer what activities really need attention from guideline developers.

### Time spent on seeking information provided by the CAN guideline

CHC professionals involved in the development of the support tool expected that using the guideline support tool would cost extra time [33]. However, the results regarding time spent seeking information provided by the CAN guideline suggest that the support tool can actually save time. The variation in time between individual professionals was large, which may explain why the difference between the intervention group (115 min) and the control group (153 min) is not significant.

### Strengths and limitations

A strength of this study is that the questionnaire sample was representative of the types of employee in the organisation. The response rate for the questionnaire was satisfactory, at 50%. Another strength of this study is that the methods allowed adherence scores to be corrected for intentional non-adherence [21]. Furthermore, the benefit of using two methods to study use of the tool and guideline adherence is that the methods compensate for each other’s biases and allow cross-validation of research findings [53].

The study is limited in four respects. First, both methods contain biases which cannot be completely excluded. The reliability of record data may be limited by poor registration performances [44]. As a result, guideline adherence may be higher than the results of the record analysis demonstrated [44]. In addition to the tendency of survey respondents to provide socially desirable answers, the reliability of self-report survey data is biased by the possibility that professionals who used the tool or find the topic interesting and important were more likely to participate. However, most adherence scores were consistent across methods, indicating reliable results.

Second, using two methods exposed contrary results regarding use of the tool and adherence to one activity, ‘Risk assessment based on protective and risk factors’, making it difficult to draw conclusions from those data.

Third, the performances of professionals in 97 out of 152 records were regarded as adherent because the record revealed that one or more professionals outside the CHC organisations performed, or were responsible for performing, guideline activities. Correcting for this in 97 records has probably led to an overestimation of adherence scores, as analysis of the other 55 cases revealed lower adherence scores for two guideline activities.

### Conclusions

As use of the support tool during the study period was low and guideline adherence rates, corrected for intentional non-adherence, were high in both study groups, no conclusions could be drawn about the effects of the tool on guideline adherence. The findings do however suggest that the tool may have the potential to save preventive child healthcare professionals’ time to seek guideline information. As this study was performed in the primary healthcare setting, the results cannot automatically be generalized to other healthcare fields.

Future research should focus on why CHC professionals accepted or did not accept the tool, using a well-regarded theory of technology acceptance such as the Technology Acceptance Model [51]. Research on socio-technical factors is considered vital to maximise the likelihood of successful implementation of digital innovations to enhance the quality of health care [25]. Also, we recommend use of an evaluation approach in which guideline deviations can easily be analysed. For example, Arts et al. [21] suggest the Standardised Clinical Assessment And Management Plan (SCAMP) for this purpose [21, 54]. We furthermore propose further development and evaluation of the tool after a longer period of availability.

Given the low number of identified cases of CAN in the record analysis, many cases of CAN are not detected by child healthcare professionals. It is therefore paramount that child healthcare organisations structurally invest in educating their professionals in recognizing CAN, to keep children safe from abuse and neglect.

## Additional file

Additional file 1 : Questionnaires for the intervention and control groups. (DOCX 36 kb)

## Data Availability

The datasets used and/or analysed during the current study are available from the corresponding author on reasonable request.

## Abbreviations

CAN: Child Abuse and Neglect
CHC: Child healthcare
IT: Information Technology
SCAMP: Standardised Clinical Assessment And Management Plan
