# Supplementary material for: Effects of a computerised guideline support tool on child healthcare professionals’ response to suspicions of child abuse and neglect: a community-based intervention trial
Source: BMC Med Inform Decis Mak. 2019 Aug 15;19:161. doi: 10.1186/s12911-019-0884-y (PMC6694693; doi:10.1186/s12911-019-0884-y)
Supplement: Supplementary file 1 — : Questionnaires for the intervention and control groups. (DOCX 36 kb) [file 12911_2019_884_MOESM1_ESM.docx]

**Additional file 1. Questionnaires for the intervention and control groups**

**QUESTIONNAIRE - INTERVENTION GROUP**

**Dear child healthcare professional,**

**Thank you for participating in this research by the Academic Collaborative Centre Youth Twente**. **Your answers will help to improve support for child healthcare professionals in handling (suspected) child abuse and neglect in the future.**

- **When answering the questions, please keep the following time-frame in mind: February 1st to October 1st 2014.**
- **This study is completely anonymous. All information in the questionnaire will be dealt with confidentially by the research team. It will not be possible for others to identify you based on your answers.**
- **You can complete the questionnaire until January 9th 2015.**

**Thank you in advance for your cooperation.**

1. **Background variables**
2. What is your position? Please select one of the following possible answers*.*

□ Child healthcare physician

□ Child healthcare nurse

1. How old are the clients you work with? Please select one of the following possible answers*.*

□ 0–4 years of age

□ 4–12 years of age

□ 12–19 years of age

□ Other: …………………………………………..

In which area do you work? Multiple answers are possible.

□ ENS

□ HOLD

□ HRHW

□ HBH

□ TAT

1. How many hours per week do you work in your position as an executive child healthcare professional?

………………………………………………… hours per week.

1. How many years have you been working as an executive child healthcare professional?

………………………………………………… years.

1. **Suspicions of child abuse/neglect**
2. For how many children in your caseload did you suspect child abuse/neglect between February 1st and November 1st 2014? Please select one of the following possible answers*.*

□ 0 children (🡪 skip questions 6-24, 26, 27)

□ 1 child

□ 2 children

□ 3 children

□ More than 3 children

1. **The computerised guideline support tool**

**The computerised guideline support tool is a computerised tool in mlCAS^[[1]](#footnote-1)^. Since February 1st 2014, you have been able to use this tool when you suspect child abuse/neglect. The computerised guideline support tool is a registration in mlCAS with the name ‘Meldcode K&HG’.**

1. Did you use the computerised guideline support tool between February 1st and October 1st 2014? Please select one of the following possible answers*.*

□ No (skip questions 26, 27)

□ Yes

**[If 6 = yes] When answering the following question, please keep the following issues in mind:**

- **The latest child you suspected was being abused and/or neglected**
- **In which you used the computerised guideline support tool**
- **Between February 1st and October 1st 2014**

**The case does not have to be completed and it is not necessary for you to retrieve information from the child’s record.**

**[If 6 = no] When answering the following question, please keep the following issues in mind:**

- **The latest child you suspected to be abused and/or neglected**
- **Between February 1st and October 1st 2014.**

**The case does not have to be completed and it is not necessary for you to retrieve information from the child’s record.**

1. Which of the following aids did you use to signal and respond to suspicions of child abuse and/or neglect?

□ The clinical guideline on child abuse and neglect prevention for preventive child healthcare (JGZ-richtlijn in Dutch)

□ The physicians’ guideline on child abuse and neglect prevention (KNMG-meldcode in Dutch)

□ The nurses’ guideline on child abuse and neglect prevention (V&VN-meldcode in Dutch)

□ The internal reporting code on child abuse and neglect prevention (De meldcode van GGD Twente in Dutch)

□ Information from the internet (except for online versions of the guidelines mentioned above)

□ None of the above

□ Other:………………………………..

1. How much time did you spend on that? Please fill in an estimate below:

…… minutes.

1. In what manner(s) did your suspicions of child abuse/neglect arise?

□ Through personal observation

□ From the story of a child

□ From the story of a fellow child healthcare professional in my organisation

□ From the story of a professional outside my organisation

□ Other:…………………………….

1. **Activities in case of suspected child abuse/neglect**

**[If 6 = yes] When answering the following question, please keep the following issues in mind:**

- **The latest child you suspected to be abused and/or neglected**
- **In which you used the computerised guideline support tool**
- **Between February 1st and October 1st 2014**

**The case does not have to be completed and it is not necessary for you to retrieve information from the child’s record.**

**[If 6 = no] When answering the following question, please keep the following issues in mind:**

- **The latest child you suspected to be abused and/or neglected**
- **Between February 1st and October 1st 2014.**

**The case does not have to be completed and it is not necessary for you to retrieve information from the child’s record.**

1. Did you assess the risk of child abuse/neglect based on protective and risk factors?

□ No

□ Yes [🡪 question 12]

1. What was your reason for not assessing the risk of child abuse/neglect based on protective and risk factors?

□ A fellow child healthcare professional in my organisation performed this activity.

□ A professional outside my organisation performed this activity

□ Other:………

1. Did you discuss your suspicions with caregiver(s) and/or the child?

□ No

□ Yes [🡪 question 14]

1. What was your reason for not discussing your suspicions with caregiver(s) and/or the child?

□ I no longer had suspicions of child abuse and/or neglect

□ I did not get to this activity before October 1st

□ A fellow child healthcare professional in my organisation performed this activity.

□ A professional outside my organisation performed this activity

□ Other:………

1. Did you consult the regional child protection service: the Advice and Reporting Centre?

□ No

□ Yes [🡪 question 16]

1. What was your reason for not consulting the regional child protection service: the Advice and Reporting Centre?

□ I no longer had suspicions of child abuse and/or neglect

□ I did not get to this activity before October 1st

□ A fellow child healthcare professional in my organisation performed this activity.

□ A professional outside my organisation performed this activity

□ Other:………

16. Did you consult the in-house CAN expert?

□ No

□ Yes [🡪 question 18]

1. What was your reason for not consulting the in-house CAN expert?

□ I no longer had suspicions of child abuse and/or neglect

□ I did not get to this activity before October 1st

□ A fellow child healthcare professional in my organisation performed this activity.

□ A professional outside my organisation performed this activity

□ Other:………

1. Did you request information from one or more professionals outside the child healthcare organisation because of your suspicions of child abuse and/or neglect?

□ No

□ Yes [🡪 question 20]

1. What was your reason for not requesting information from one or more professionals outside the organisation?

□ I no longer had suspicions of child abuse and/or neglect

□ I did not get to this activity before October 1st

□ A fellow child healthcare professional in my organisation performed this activity.

□ A professional outside my organisation performed this activity

□ The caregiver(s) or the child did not approve

□ Other:………

1. Did you act on your suspicions of CAN, e.g. by reporting to the Advice and Reporting Centre, referring, or providing support?

□ No [🡪 skip questions 22 and 23]

□ Yes [🡪 question 22]

1. What was your reason for not acting on of your suspicions of CAN, e.g. by reporting to the Advice and Reporting Centre, referring, or providing support?

□ I no longer had suspicions of child abuse and/or neglect

□ I did not get to this activity before October 1st

□ A fellow child healthcare professional in my organisation performed this activity

□ A professional outside my organisation performed this activity

□ Other:………

1. Did you check whether support was provided to the family?

□ No

□ Yes [🡪 question 24]

1. What was your reason for not checking whether support was provided to the family?

□ I no longer had suspicions of child abuse and/or neglect

□ There was no report or referral

□ A fellow child healthcare professional in my organisation performed this activity.

□ A professional outside my organisation informed me about initiating the support.

□ Other:………

1. If you wish to clarify one or more questions, please use the field below.

…………………………………………………………………………………………………

…………………………………………………………………………………………………

1. **Familiarity with the tool**
2. Are you familiar with the computerised guideline support tool?

□ I don’t know about the computerised guideline support tool

□ I am aware of the computerised guideline support tool but did not read the manual or test the computerised guideline support tool that was available in a test environment.

□ I am aware of the computerised guideline support tool and read the manual or the computerised guideline support tool, which was available in a test environment, superficially.

□ I am aware of computerised guideline support tool and read the manual or the computerised guideline support tool, which was available in a test environment, thoroughly.

**(F) Time spent on using the computerised guideline support tool**

1. How much time did you spend on learning to use the computerised guideline support tool?

………….. minutes.

1. Between February 1st and October 1st 2014 how much time did you spend using the computerised guideline support tool? When answering the following question, please think about case of the latest child you suspected was being abused and/or neglected in which you used the computerised guideline support tool.

………….. minutes.

1. If you would like to write down any remarks with regard to the questionnaire, please use the field below.

……………………………………………….………………………………………………………………………………………………………………………………………………………………………………………………………………………………………………………………………………………………………………………………

**Thank you for your participation.**

**QUESTIONNAIRE – CONTROL CONDITION**

**Dear child healthcare professional,**

**Thank you for participating in this research by the Academic Collaborative Centre Youth Twente. Your answers will help to improve support for child healthcare professionals in handling (suspected) child abuse and neglect in the future.**

- **When answering the questions, please keep the following time-frame in mind: February 1st to October 1st 2014.**
- **This study is completely anonymous. All information in the questionnaire will be dealt with confidentially by the research team. It will not be possible for others to identify you based on your answers.**
- **You can complete the questionnaire until January 9th 2015.**

**Thank you in advance for your cooperation.**

1. **Background variables**
2. What is your position? Please select one of the following possible answers*.*

□ Child healthcare physician

□ Child healthcare nurse

1. How old are the clients you work with? Please select one of the following possible answers*.*

□ 0–4 years of age

□ 4–12 years of age

□ 12–19 years of age

□ Other: …………………………………………..

In which area do you work? Multiple answers are possible.

□ ENS

□ HOLD

□ HRHW

□ HBH

□ TAT

1. How many hours per week do you work in your position as an executive child healthcare professional?

………………………………………………… hours per week.

1. How many years have you been working as an executive child healthcare professional?

………………………………………………… years.

1. **Suspicions of child abuse/neglect**
2. For how many children in your caseload did you suspect child abuse/neglect between February 1st and November 1st 2014? Please select one of the following possible answers*.*

□ 0 children (🡪 skip questions 6-24, 26, 27)

□ 1 child

□ 2 children

□ 3 children

□ More than 3 children

1. **Activities in case of suspected child abuse/neglect**

**When answering the following question, please keep the following issues in mind:**

- **The latest child you suspected was being abused and/or neglected**
- **Between February 1st and October 1st 2014.**

**The case does not have to be completed and it is not necessary for you to retrieve information from the child’s record.**

1. Which of the following aids did you use to signal and respond to suspicions of child abuse and/or neglect?

□ The clinical guideline on child abuse and neglect prevention for preventive child healthcare (JGZ-richtlijn in Dutch)

□ The physicians’ guideline on child abuse and neglect prevention (KNMG-meldcode in Dutch)

□ The nurses’ guideline on child abuse and neglect prevention (V&VN-meldcode in Dutch)

□ The internal reporting code on child abuse and neglect prevention (De meldcode van GGD Twente in Dutch)

□ Information from the internet (except for online versions of the guidelines mentioned above)

□ None of the above

□ Other:

1. How much time did you spend on that? Please fill in an estimate below:

…… minutes.

1. In what manner(s) did your suspicions of child abuse/neglect arise?

□ Through personal observation

□ From the story of a child

□ From the story of a fellow child healthcare professional in my organisation

□ From the story of a professional outside my organisation

□ Other:…………………………………

1. Did you assess the risk of child abuse/neglect based on protective and risk factors?

□ No

□ Yes [🡪 question 11]

1. What was your reason for not assessing the risk of child abuse/neglect based on protective and risk factors?

□ A fellow child healthcare professional in my organisation performed this activity.

□ A professional outside my organisation performed this activity

□ Other:………

1. Did you discuss your suspicions with caregiver(s) and/or the child?

□ No

□ Yes [🡪 question 13]

1. What was your reason for not discussing your suspicions with caregiver(s) and/or the child?

□ I no longer had suspicions of child abuse and/or neglect

□ I did not get to this activity before October 1st

□ A fellow child healthcare professional in my organisation performed this activity

□ A professional outside my organisation performed this activity

□ Other:………

1. Did you consult the regional child protection service: the Advice and Reporting Centre?

□ No

□ Yes [🡪 question 15]

1. What was your reason for not consulting the regional child protection service: the Advice and Reporting Centre?

□ I no longer had suspicions of child abuse and/or neglect

□ I did not get to this activity before October 1st

□ A fellow child healthcare professional in my organisation performed this activity

□ A professional outside my organisation performed this activity

□ Other:………

15. Did you consult the in-house CAN expert?

□ No

□ Yes [🡪 question 17]

16. What was your reason for not consulting the in-house CAN expert?

□ I no longer had suspicions of child abuse and/or neglect

□ I did not get to this activity before October 1st

□ A fellow child healthcare professional in my organisation performed this activity

□ A professional outside my organisation performed this activity

□ Other:………

1. Did you request information from one or more professionals outside the child healthcare organisation because of your suspicions of child abuse and/or neglect?

□ No

□ Yes [🡪 question 19]

1. What was your reason for not requesting information from one or more professionals outside the organisation?

□ I no longer had suspicions of child abuse and/or neglect

□ I did not get to this activity before October 1st

□ A fellow child healthcare professional in my organisation performed this activity

□ A professional outside my organisation performed this activity

□ The caregiver(s) or the child did not approve.

□ Other:………

1. Did you act on your suspicions of CAN, e.g. by reporting to the Advice and Reporting Centre, referring, or providing support?

□ No [🡪 skip questions 21 and 22]

□ Yes [🡪 question 21]

1. What was your reason for not acting on your suspicions of CAN, e.g. by reporting to the Advice and Reporting Centre, referring, or providing support?

□ I no longer had suspicions of child abuse and/or neglect

□ I did not get to this activity before October 1st

□ A fellow child healthcare professional in my organisation performed this activity.

□ A professional outside my organisation performed this activity

□ Other:………

1. Did you check whether support was provided to the family?

□ No

□ Yes [🡪 question 23]

1. What was your reason for not checking whether support was provided to the family?

□ I no longer had suspicions of child abuse and/or neglect

□ There was no report or referral.

□ A fellow child healthcare professional in my organisation performed this activity.

□ A professional outside my organisation informed me about initiating the support.

□ Other:………

1. If you wish to clarify one or more questions, please use the field below.

…………………………………………………………………………………………………

1. If you would like to write down any remarks with regard to the questionnaire, please use the field below.

…………………………………………………………………………………………………………………………

**Thank you for your participation.**

1. mlCAS is the name of the electronic child health record system used by child healthcare professionals working at the municipal health service GGD Twente. [↑](#footnote-ref-1)
